# Immune response and stuttering: the potential link between cytokines and stutter

**DOI:** 10.1016/j.bjorl.2026.101774

**Published:** 2026-07-27

**Authors:** Saime Güzelsoy Sagiroglu, Metin Kılınc, Israfil Orhan, Omer Faruk Cinar, Muhammed Gazi Yıldız, Adem Doganer, Emine Elif Altuntas

**Affiliations:** a Kahramanmaras Sutcu Imam University, Faculty of Medicine, Department of Otorhinolaringology, Kahramanmaras, Turkey; b Kahramanmaras Sutcu Imam University, Faculty of Medicine, Department of Biochemisty, Kahramanmaras, Turkey; c Kahramanmaraş Sutcu Imam University, Faculty of Medicine, Department of Biostatistics and Medical Informatics, Kahramanmaras, Turkey; d Lokman Hekim University, Faculty of Medicine, Department of Otorhinolaringology, Ankara, Turkey

**Keywords:** Stuttering, Cytokine, Pediatric, Immune system, Etiology

## Abstract

**Objective:**

Stuttering is a common speech disorder among the general population, characterized by disruptions in the fluency of speech. Even if many studies have been managed on stuttering, the etiology has not yet been exactly explained. This article aims to assess the level of cytokines in patients who stutter to provide evidence-based guidelines for clinicians.

**Methods:**

A total of 74 children, including 34 who were stutterers and 40 who were controls, aged 4–15 years were included. The children with stuttering completed the Stuttering Severity Instrument-3 (SSI-3) form. Serum concentrations of IL-1, IL-6, IL-8, and TNF-α were analyzed in blood samples obtained from the subjects.

**Results:**

Of the stuttering children we included in the study, 3 (8.8%) had very mild, 13 (38.2%) had mild, (35.2%) had moderate, 4 (11.7%) had severe, and 2 (5.8%) had very severe stuttering. IL-1, IL-8, and TNF-α levels in stuttering patients were significantly higher compared to the control group (p = 0.028, p = 0.031, p < 0.001, respectively). The IL-6 level did not show a statistically significant difference between the groups (p = 0.461). Regarding the diagnostic performance of stuttering, IL-1, IL-6, IL-8, and TNF-α all demonstrated statistically significant ability to distinguish between patients and the control group (p = 0.025, p = 0.021, p = 0.004, p = 0.003, respectively).

**Conclusions:**

These results suggest an increase in circulating levels of cytokines known to be regulatory and that stuttering is associated with a proinflammatory state. These results may be well-considered important in the diagnosis and evaluation of stuttering. This study contributes further support to investigate the immune system as a target for future therapeutic development.

**Level of evidence:**

Level 4.

## Highlights

• This study is the first to propose a neuroinflammation hypothesis for stuttering.
• Serum IL-1, IL-8, and TNF-α levels were significantly higher in stuttering children.
• The immune system may have a potential role in the pathophysiology of stuttering.

## Introduction

Stuttering is a speech fluency disorder, characterized by repetition, prolongation, and blocks of syllables, words, or phrases. Stuttering is not easily controlled and may be associated with other negative emotions and movements, such as fear, shame, or nervousness. While stuttering is technically a symptom rather than a disease, the term is often used to refer to both the condition and the symptom.^1^ Around 1% of the general population experiences stuttering and this ratio rises to 8 %–11% in children.^2^ This condition is four times more common in males than in females. Stuttering is classified into two types: acquired and developmental. Acquired stuttering may stem from neurogenic, psychogenic, pharmacogenic, or iatrogenic causes.^3^ Developmental stuttering, the most common form, begins in childhood and can persist into adulthood. Studies have reported that stuttering is a neurodevelopmental motor disease, although genetic factors have also been held responsible for the development of the disease.^4^ However, the underlying neurological arrangements are still being debated. In addition to genetic components, there are also significant nongenetic factors.^5^ It has also been suggested that an inflammatory process could play a role in the etiopathogenesis.^6^

Recent research demonstrates cytokines, which regulate immune and inflammatory processes such as cell growth, healing, and systemic responses to injury, may play a significant role in neurological and psychiatric conditions.^7^^,^^8^ Although cytokines are known to be associated with immune response and inflammation, studies have shown that they may also be associated with bacterial septic shock, some types of cancer, Chagas disease and neurological diseases.^9^, ^10^, ^11^, ^12^ Similarly, the results of meta-analysis studies reveal the levels of IL-1, IL-6, IL-8, and TNF-α increase, which are among the systemic inflammation markers, in depression, fear, and anxiety disorders.^13^, ^14^, ^15^ These findings suggest that cytokines, with special interest on IL-1, IL-6, IL-8, and TNF-α, could influence the pathophysiology of stress-related disorders through their impact on inflammatory and immune pathways. Given that stuttering often induces stress and anxiety in affected individuals, these cytokines may have a plausible role in modulating the severity or progression of stuttering.^16^^,^^17^

In conclusion, serum cytokine panels are increasingly utilized in medical practice, yet the comprehension of their role as disease biomarkers remains limited. Although there is no definitive evidence to date showing that cytokines and inflammatory processes may have a direct or indirect connection with stuttering, we thought that there may be a relationship, just like in neurological disorders and psychiatric conditions, and we proposed the null hypothesis, “Does the increase in the levels of systemic inflammatory markers IL-1, IL-6, IL-8, and TNF-α have a role in the pathophysiology of stuttering?”. Our study aimed to investigate whether IL-1, IL-6, IL-8 and TNF-α, all important modulators of the immune response and are included in the cytokine panel we use in our routine clinical practice, play a part in the development of stuttering cases observed in the pediatric age group.

## Methods

### Study population and design

This prospective, case–control study was carried out in the Ear, Nose, and Throat Clinic of University, Faculty of Medicine. The study includes a total of 74 children, with 34 patients and 40 in the control group. The patients were those who visited the ENT ‒ Phoniatrics Clinic due to stuttering and those with developmental stuttering. The control group included children who applied to the pediatric polyclinic for a health check and had no speech disorders. The control group included children in the 5–15 years age range, who had the same age and gender distribution as the patient group and no stuttering diagnosis.

Inclusion criteria: (1) Being aged 4–15 years-old; (2) Being diagnosed with stuttering by a specialist doctor according to DSM-V criteria^18^; (3) Speaking Turkish as the native language; (4) Suffering from developmental stuttering.

Exclusion criteria: (1) Neurological conditions and trauma-induced stuttering (acquired stuttering); (2) Children affected by psychiatric disorders, including ADHD, MDD, and social anxiety disorder; (3) Neurological, metabolic, or chronic conditions, organic pathologies that could impair speech fluency (such as missing some or all of the incisor teeth); (4) History of drug use; (5) Children exposed to smoking; (6) Children had any systemic disease, a recent history of surgery under general anesthesia, the development of pathology in the respiratory tract due to consumption of corrosive substances for different reasons; (7) The presence of active infection and had no history of URTI in the past 4-weeks; (8) Malignancy, or a history of radiotherapy to the head and neck area.

The research procedure was first explained thoroughly to children and their parents. The participants were enrolled in the study confirming their correspondence with the inclusion criteria. All study procedures were conducted in compliance with the Helsinki Declaration. The parents or legal guardians of each child had given their informed consent.

### Instrument to measure stuttering severity

The Stuttering Severity Assessment Scale 3 (SSI-3), developed by Riley in 1994, is used to assess the severity of stuttering. It is designed to evaluate stuttering severity in both adults and children. The frequency, duration and physical accompaniments of stuttering are evaluated in three separate categories and a score is obtained for the severity of stuttering by summing the scores obtained. A total score between 0 and 10 indicates very mild, 11 and 16 indicates mild, 17 and 26 indicates moderate, 27 and 31 indicates severe and 32 and above indicates very severe stuttering.^19^

### Biochemical analysis

For each subject, blood samples were collected in Vacutainers containing acid-citrate dextrose (BD Biosciences; San Jose, CA). The samples were centrifuged at 2300 rpm for 10-minutes to separate the plasma. The collected plasma was stored at −80 °C until cytokine analysis was performed on the day the samples were first thawed. The plasma samples were processed according to the instructions of the kit protocol.

### Statistical analysis

The normality of the variables was assessed using the Shapiro-Wilk test. For variables not following a normal distribution, analysis made using the Mann-Whitney *U* test. The association between categorical variables was analyzed using the Chi-Square and Fisher's Exact test. ROC analysis was performed to evaluate the diagnostic performance of biochemical variables for the disease. Differences in IL-8, IL-6, IL-1, and TNF α values across different degrees of stuttering were analyzed using the Kruskal-Wallis H test, with post-hoc tests conducted using the Dunn-Sidak method. Statistical parameters were reported as median (Q1‒Q3) values or number (n) and percentage (%). A p-value of < 0.05 was considered statistically significant. The data collected in the study were analyzed using IBM SPSS version 22 and R 3.3.2.

## Results

The patient group comprised 88.2% (n = 30) males and 11.76% (n = 4) females. The average age was 8.93 ± 3.87 years (min‒max age: 4‒15 year). The control group comprised of 67.5% (n = 27) males and 32.5% (n = 13) females with a mean age of 8.73 ± 3.21 years (min‒max: 5–15 years). The age of onset of stuttering was 4.1 ± 1.4 years and the average duration of stuttering in the patient group was 5.5 ± 2.3 years (range: 6-months‒10-years). A familial history of stuttering was identified in 32.3% (n = 11) children, 26.4% (n = 9) had a background of rapid speech, and 47.0% (n = 16) had a background of fear-initiated stuttering.

**Fig. 1:**
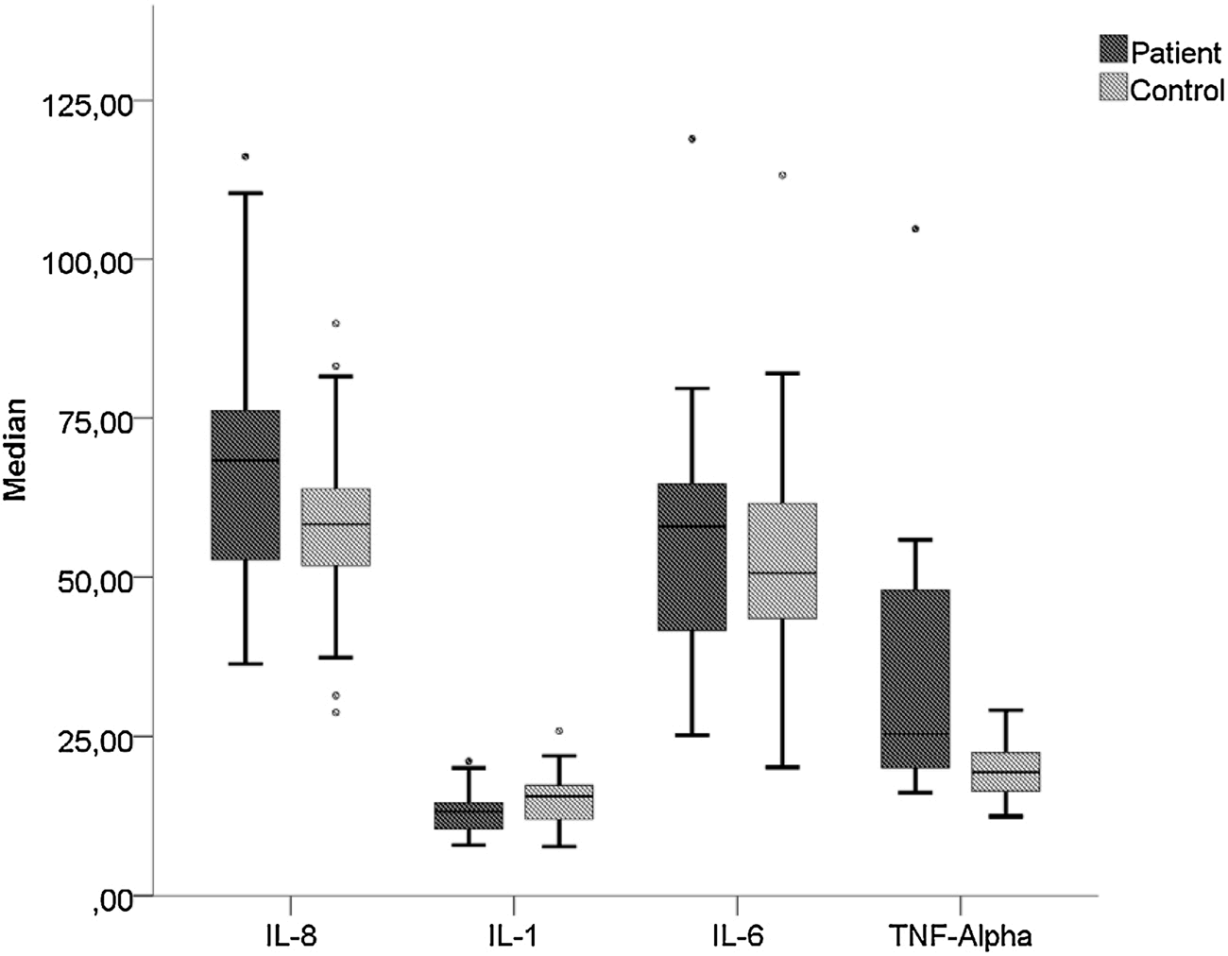
Evaluation of IL-1, IL-6, IL-8 and TNF-α activity in the patient and control group with a box plot curve.

The IL-1, IL-6, IL-8, and TNF-α activities of the stuttering and control groups are presented with box plot curves in Fig. 1, and the levels are also provided in Table 1. The IL-8 and TNF-α levels in the stuttering patients were found to be higher than the control group, with this difference being statistically significant (p = 0.031; p < 0.001). IL-1 level showed a higher IL-1 level in the control group compared to stuttering patients and this difference was deemed statistically relevant (p = 0.028).

**Table 1:** IL-1, IL-6, IL-8 and TNF-α levels in stuttering and control group.

		Patient	Control	p
Variable 002	Median (Q1‒Q3)	0.3208 (0.2477‒0.3576)	0.2739 (0.2435‒0.3000)	0.031
IL 8	Median (Q1‒Q3)	68.32 (52.76–76.17)	58.34(51.87–63.90)	0.031
IL 1	Median (Q1‒Q3)	13.28 (10.48–14.60)	15.70(12.04–17.34)	0.028
IL 6	Median (Q1‒Q3)	57.99 (41.67–64.64)	50.68(43.60–61.64)	0.461
TNF-α	Median (Q1‒Q3)	25.37 (20.07–48.05)	19.40(16.44–22.54)	p < 0.001

**Fig. 2:**
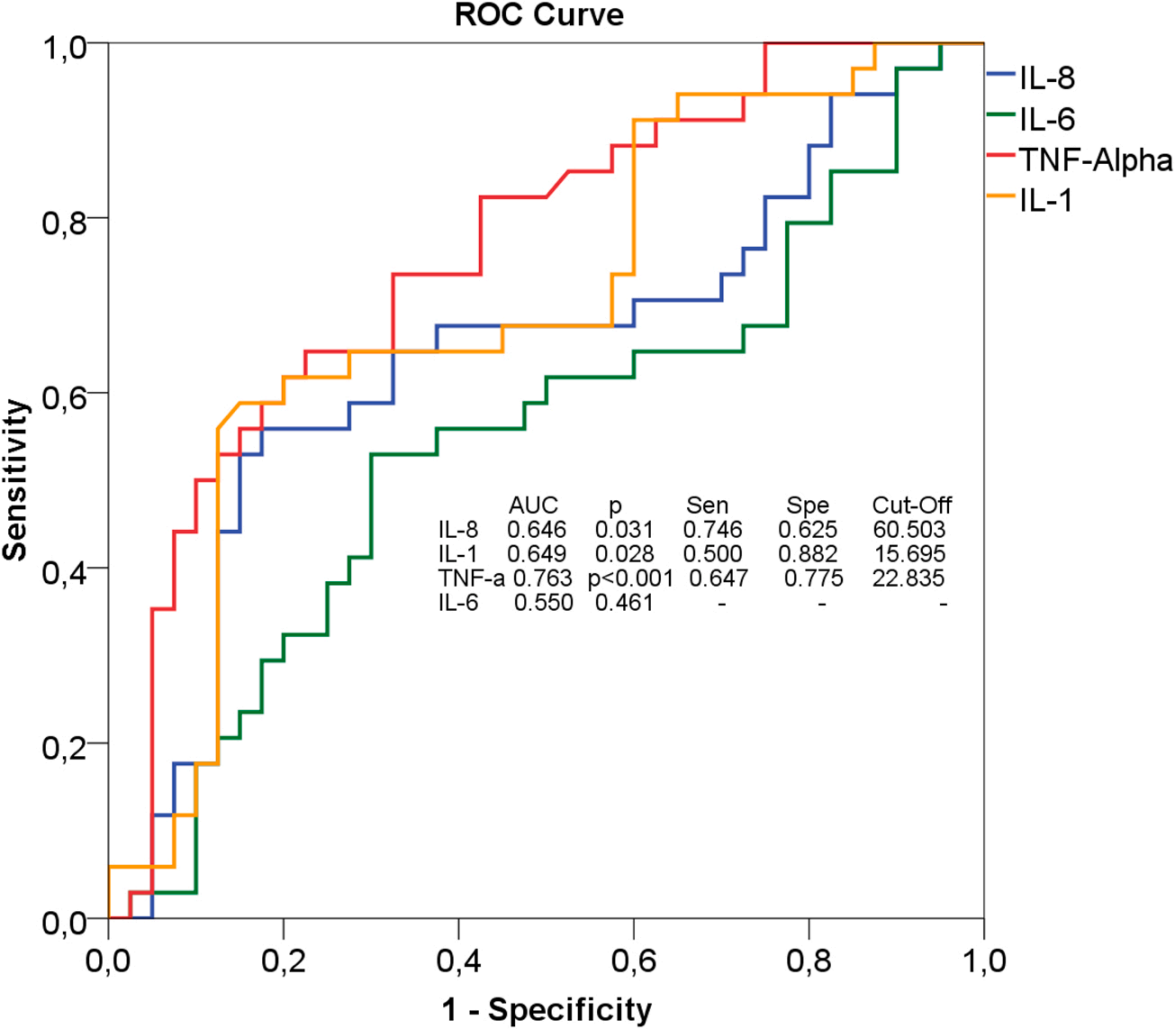
ROC curve of IL-1, IL-6, IL-8 and TNF-α of patients and control group.

ROC curve of IL-1, IL-6, IL-8 and TNF-α of patients and control group are shown in Fig. 2. In the ROC analysis, the cut-off points (AUC) for IL-1, IL-8, and TNF-α were calculated as 0.649, 0.646, and 0.763, in this order, and the differences were found to be statistically significant (p = 0.028; p = 0.031; p < 0.001). High values indicated stuttering for TNF-α and IL-8, and with cutoff values of 22.835 and 60.503, the sensitivity and specificity values were 64.7% and 77.5% for TNF-α, and 74.6% and 62.5% for IL-8, respectively. For IL-1, low values were indicative of stuttering, with a cutoff point of 15.695, resulting in a sensitivity of 50.0% and a specificity of 88.2%. IL-1, IL-8, TNF-a were found to be definite markers in stuttering patients. Therefore, it was determined that these values had high specificity and positive predictive value. The area under the ROC curve was found to be not statistically significant for IL-6 at 0.550 (p = 0.461).

The mean SSI-3 score used to determine the severity of stuttering was 22.6 ± 12.1. In assessing stuttering severity, 8.8% (n = 3) of patients were classified as very mild, 38.2% (n = 13) as mild, 35.2% (n = 12) as moderate, 11.7% (n = 4) were listed severe and 5.8% (n = 2) were considered very severe.

**Fig. 3:**
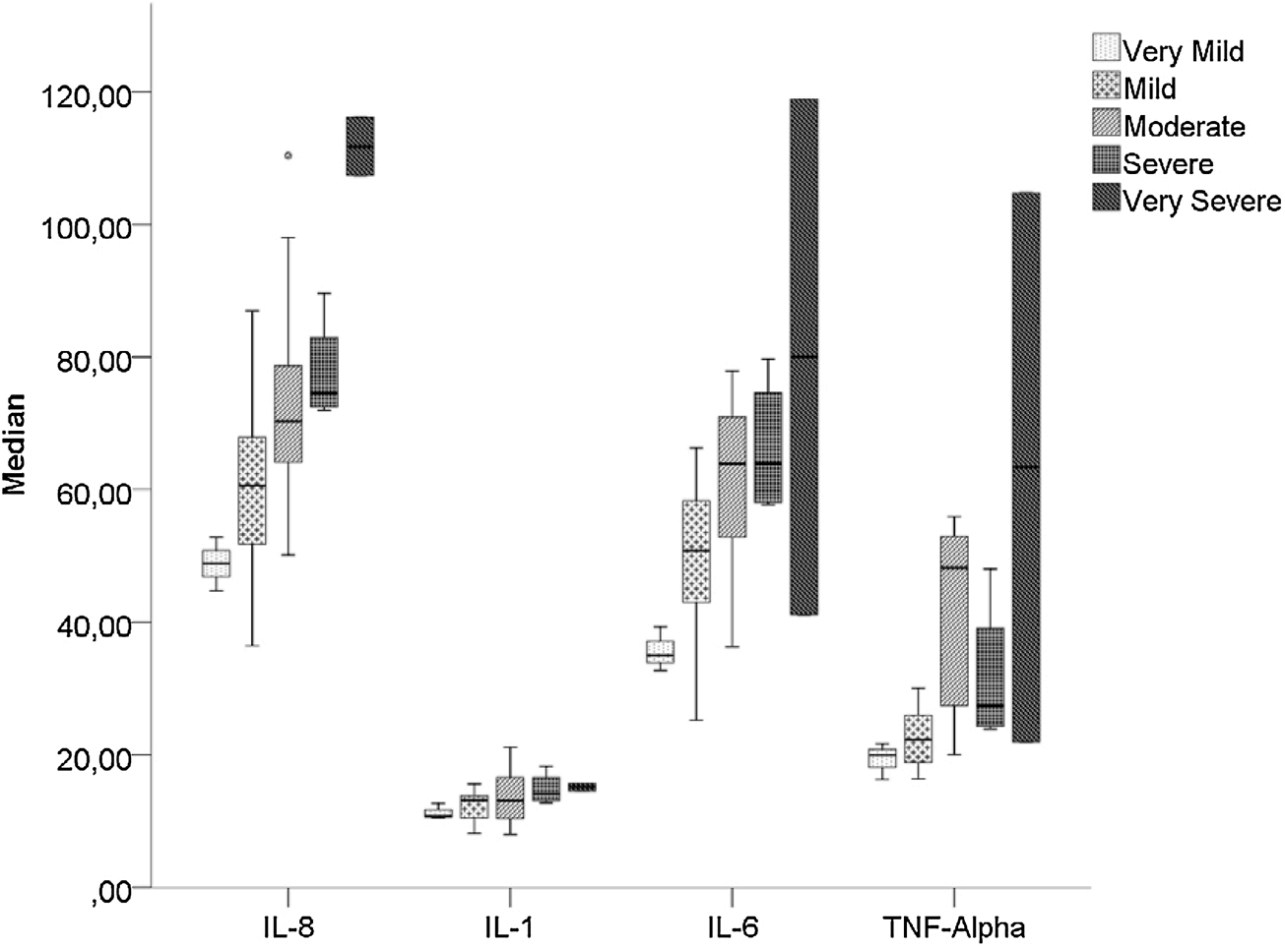
Evaluation of IL-1, IL-6, IL-8 and TNF-α activity in patient and control group according to the severity of stuttering using box plot curve.

Table 2 displays the IL-1, IL-6, IL-8 and TNF-α activity values based on the severity of the stutter. The difference between stuttering degrees in terms of IL-1, IL-6, IL-8, and TNF-α levels was determined as statistically significant (respectively; p = 0.025, p = 0.021, p = 0.004 and p = 0.003). As stuttering severity increases, IL-1, IL-6, IL-8 and TNF-α levels also rise (Fig. 3).

**Table 2:** IL-1, IL-6, IL-8 and TNF-α levels according to the severity of the stuttering.

	SSI Degree (n = 34)					
	Very Mild	Mild	Moderate	Severe	Very Severe	p
IL_1	10.73 (10.48–12.72)	13.18 (10.47–13.80)	13.08 (10.37–16.58)	14.15 (13.12–16.59)	15.14 (14.60–15.68)	0.025*
IL_6	34.99 (32.72–39.33)	50.78 (42.99–58.26)	63.88 (52.78–70.93)^a^	63.92 (58.05–74.59)	80.03 (41.14–118.92)	0.021*
IL_8	48.86 (44.75–52.76)	60.60 (51.76–67.84)	70.28 (64.09–78.65)	74.54 (72.44–82.91)	111.76 (107.37–116.15)^a^	0.004*
TNF-α	19.94 (16.25–21.68)	22.27 (18.86–25.97)	48.20 (27.44–52.91)^a,b^	57.45 (24.34–39.09)	63.37 (21.96–104.78)	0.003*

Kruskal Wallis H test; a = 0.05; Dunn sidak test.

* The difference between the groups is significant; the difference with the ^a^ Very mild group is significant; the difference with the ^b^ Mild group is significant; the difference with the ^c^ Moderate group is significant; the difference with the ^d^ Severe group is significant; the difference with the ^e^ Very severe group is significant.

## Discussion

Stuttering is a common but poorly understood disease. In recent years, studies investigating cytokines and inflammation mechanisms in the origin of neurological and psychiatric conditions have been rapidly increasing. In this study, the neuroinflammation theory, believed to have a role in the emergence of stuttering, was used as a basis, and the serum levels of certain systemic inflammatory markers were evaluated in pediatric cases to investigate the potential of inflammatory processes driven by cytokines in the pathophysiology of stuttering. Our findings indicate that IL-6, IL-8, and TNF-α values are higher, while IL-1 levels were decreased in children who stutter. We also showed that cytokine levels increase in parallel with the increase in stuttering severity. However, the clinical significance and potential use of these cytokine level changes as biomarkers remain inconclusive, highlighting the need for further research with larger cohorts.

Many theories have been proposed, and many studies have been conducted about the etiology of stuttering. One of the significant aspects emphasized in terms of etiology is the neuroinflammation theory. Ischemia-reperfusion injury occurring in the frontal region, especially Broca's area and basal ganglia, which is thought to begin in the perinatal period and is provoked by the process of childbirth, together with chronic inflammation and consequent neuromotor disorder is one of the important theories implicated in stuttering.^8^ The basal ganglia-thalamocortical motor circuits, including the putamen, may have a significant role in stuttering.^20^^,^^21^ Studies suggest that acquired stuttering is caused by a lesion in this circuit, while the primary speech and language centers, including Broca's area, planum temporale, insula, and Wernicke's area, remain intact.^22^ Another etiological factor under consideration is changes in biochemical parameters. Significant research has been conducted to examine the bioelement levels in samples from patients with neurological and psychological disorders. Magnesium, calcium, and copper concentrations have been studied in the blood and hair samples of stuttering patients; however, the results of these studies have not shown consistency with each other.^23^^,^^24^ Based on these publications, it appears to be an undeniable fact that inflammation may also play a potential role in stuttering cases, whose etiology remains unclear. However, no studies investigating cytokine levels in stuttering patients were found in the available databases. Therefore, we aimed to determine whether IL-1, IL-6, IL-8, and TNF-α, which are included in the cytokine panel routinely used in our clinical practice, contribute to the development of stuttering cases observed in the pediatric age group.

Initially identified as products of immune system cells that regulate immune processes, it is now understood that many cytokines are also produced by non-immune system cells and can influence those cells as well. One of these cytokines is IL-1, which is essential for regulating the body's immune and inflammatory responses, to maintain homeostasis and provide protection against pathogens and tissue damage. IL-1 is the primary endogenous pyrogen that triggers fever through the hypothalamus.^25^ Elevated IL-1 production is linked to several diseases, including Alzheimer's disease, trauma, hemodialysis, ischemic myocardial infarction, asthma, and graft-versus-host disease.^26^ Although IL-1 is essential for supporting effective immune responses, dysregulated IL-1 signaling may contribute to chronic inflammatory conditions and autoimmune diseases. Our study, the IL-1 level was found to be statistically significantly decreased in the stuttering patients when compared to the control group. Interestingly, IL-1, which increased in parallel with the severity of stuttering, has been shown to be associated with stuttering. In addition, the IL-1 was found to be a statistically significant biomarker in the diagnosis of stuttering and distinguishing the control group.

IL-6 is a cytokine with multiple functions, that has critical roles in inflammation, immunity, hematopoiesis, and tissue homeostasis, with effects on health and disease. Dysregulation of IL-6 has a role in several diseases, including autoimmune disorders, chronic inflammatory conditions, and cancer. IL-6 may also display anti-inflammatory effects.^27^^,^^28^ Numerous studies have revealed that dysregulated IL-6 production in synovial membrane cells of rheumatoid arthritis, myeloma cells, enlarged lymph nodes in Castleman’s disease, peripheral blood cells, associated tissues in a range of autoimmune diseases and chronic inflammatory conditions, and even cancerous cells in malignancies.^9^^,^^10^^,^^29^^,^^30^ In our study, although it was found to be higher in patients who stutter, it was not found to be statistically significant. We believe that this IL-6 cytokine may also be significant when studies with larger sample sizes are conducted. Because we found in this study that IL-6 increases in direct proportion to the severity of stuttering.

IL-8 is a chemokine, a signaling protein that facilitates the immune system's response to inflammation and infection. IL-8 is secreted primarily by cells such as macrophages, epithelial cells, and endothelial cells in response to a variety of stimuli, including pro-inflammatory cytokines and microbial products.^31^^,^^32^ There are various studies in the literature showing that IL-8 is elevated in inflammatory conditions.^33^^,^^34^ Dysregulation of IL-8 signaling has been shown to play a role in several inflammatory diseases, including inflammatory bowel disease and certain types of cancer. Our study results showed that IL-8 levels were statistically significantly higher in the patient group, indicating that understanding the regulation and functions of IL-8 is crucial for developing treatment strategies for stuttering. Additionally, we found that IL-8 was a statistically meaningful biomarker in diagnosing stuttering patients and differentiating them from the control group.

The primary function of TNF is to regulate immune cells. It stimulates the expression of adhesion molecules, the secretion of chemokines by macrophages, the apoptosis of target cells, and leads to systemic effects such as fever. TNF-α expression and TNF protein production are observed in most cancer biopsies. Reports indicate that TNF mRNA or TNF protein is found in 50%–70% of ovarian and breast cancers.^35^ It is the primary cytokine that activates platelets and contributes to the development of fever, anemia, and cachexia.^36^ TNF-α can also increase IL-8 production by inflamed mucosa. A previous study stated that the mRNA measurements of both TNF-α and IL-8 in the mucosa decreased in parallel with the decrease in local inflammation after the successful eradication of *H. pylori*.^36^ In the current study, high TNF-α levels were determined especially in the stuttering child patient group and it was found to be a statistically significant biomarker in distinguishing stuttering disease. In addition, the increase of both IL-8 and TNF-α in parallel with the severity of stuttering suggests that our study is a valuable finding supporting the neuroinflammation theory. In addition, the ROC analysis supports that these cytokines are important biomarkers that distinguish stuttering and can be an important guiding biomarker for future studies.

The primary limitation of our study is the limited sample size. We only examined the cytokines that are examined in routine practice. If the cytokine parameters could have been increased and examined in a wider range, we could have brought a different perspective to this study. If iron, copper, magnesium and zinc had also been examined in addition to cytokines, this study could have provided very valuable information to the literature. In addition, the fact that these profiles were not evaluated separately in terms of gender is another limitation of our study. However, this is the first study in the literature that examined cytokine levels in stuttering patients. Therefore, we think that these results provide a different perspective on the etiology of stuttering. In addition, we think that we have drawn attention to the necessity of investigating the underlying inflammation and autoimmunity in stuttering patients who have failed treatment with this study. If this situation is clarified in the future, we hypothesize that it could open a new area in medical treatment.

## ORCID ID

Metin Kılıc: 0000-0002-7384-0997

Adem Doganer: 0000-0002-0270-9350

Israfil Orhan: 0000-0002-9557-7050

Ömer Faruk Çınar: 0000-0001-5281-1620

Muhammed Gazi Yıldız: 0000-0002-1880-0685

Emine Elif Altuntas: 0000-0003-4503-3730

## Conclusion

This study explores the potential involvement of neuroinflammation in the etiology of stuttering by evaluating IL-1, IL-6, IL-8, and TNF-α levels in pediatric patients. Our findings suggest that these cytokines may exhibit differences in individuals with stuttering. Given the multifactorial nature of stuttering, further large-scale, longitudinal studies are needed to better understand the role of cytokines in its pathophysiology. Nonetheless, as one of the first studies to evaluate cytokine levels in pediatric stuttering and to propose an innovative hypothesis linking the neuroinflammation theory with immune response mechanisms, we believe that our findings provide a valuable contribution to the literature.

## CRediT authorship contribution statement

The authors confirm contribution to the paper as follows: study conception and design: SGS, EEA, IO; data collection: SGS, OFC, IO; analysis and interpretation of results: SGS, MGY, AD; draft manuscript preparation: SGS, MK, EEA, MGY. All authors reviewed the results and approved the final version of the manuscript.

## Ethics committee approval

This study was approved by the local Medical Research Ethics Committee (date: 13.07.2018, session nº 2018/12, decision nº 04).

## Funding

In this study, the remaining biochemical materials from the Kahramanmaras Sutcu Imam University Scientific Research Project (Project number: 2018/2-47M) were used. Therefore, we would like to thank Kahramanmaras Sutcu Imam University Scientific Research Project Department.

## Availability of data

Data available on request due to privacy/ethical restrictions.

## Data availability statement

The authors declare that all data are available in repository.

## Declaration of competing interest

The authors declare no conflicts of interest.
